# Comparison of *in vivo* Adjuvanticity of Liposomal PO CpG ODN with Liposomal PS CpG ODN: Soluble *Leishmania* Antigens as a Model

**Published:** 2012

**Authors:** Ensieh Golali, Mahmoud Reza Jaafari, Ali Khamesipour, Azam Abbasi, Zahra Saberi, Ali Badiee

**Affiliations:** 1 *Nanotechnology Research Centre, School of Pharmacy, Mashhad University of Medical Sciences, Mashhad, Iran*; 2 *Biotechnology Research Centre, School of Pharmacy, Mashhad University of Medical Sciences, Mashhad, Iran*; 3 *Centre for Research and Training in Skin Diseases and Leprosy, Tehran University of Medical Sciences, Tehran, Iran*

**Keywords:** CpG ODNs, Leishmaniasis, Liposome, SLA, Vaccine

## Abstract

**Objective(s):**

CpG oligodeoxynucleotides (CpG ODNs) have been shown to have potent immunostimulatory adjuvant activity for a wide range of antigens. Due to susceptibility of phosphodiester CpG ODNs (PO CpG) to nuclease degradation, nuclease-resistant phosphorothioate CpG ODNs (PS CpG) were currently utilized in an *in vivo* model. In this study, according to some recently reported drawbacks with PS CpG, the adjuvant potential of liposomal PO CpG as a substitute for PS CpG was evaluated.

**Materials and Methods:**

Soluble *Leishmania* antigens (SLA) as a model antigen and distearoylphosphatidylcoline (DSPC) as a neutral lipid were employed to prepare liposomes. Susceptible BALB/c mice received buffer, SLA, Lip-SLA, Lip-SLA-PS CpG, Lip-SLA-PO CpG, SLA+PS CpG, or SLA+PO CpG subcutaneously 3 times with 3 weeks intervals and then were challenged with *Leishmania* major’s live promastigotes. Blood and spleen samples were analyzed to determine the level and type of antibodies and cytokines. The number of live parasites in the spleen of immunized mice was determined. Moreover, the lesion size progress was assessed weekly by footpad swelling measurement.

**Results:**

The results showed that mice immunized with Lip-SLA-PS CpG or Lip-SLA-PO CpG developed a significantly smaller footpad swelling, higher level of anti SLA IgG antibodies before and after challenge, and lower spleen parasite burden compared with the control groups. However, there was no significant difference between mice received Lip-SLA-PS CpG and those received Lip-SLA-PO CpG.

**Conclusion:**

The results demonstrated that liposomal PO CpG ODN could be used instead of PS CpG ODN to overcome the possible drawbacks.

## Introduction

Cytosine phosphate guanine oligodeoxynucleotides (CpG ODNs) are DNA motifs with high frequency in bacteria, so mimic the immunostimulatory activity of bacterial DNA (1, 2). They make improvement in Ag uptake, presentation by antigen presenting cells (APCs) and antibodies, chemokines, and cytokines secretion by B cells, natural killer (NK) cells, dendritic cells (DCs), and monocytes *via* interaction with toll-like receptor 9 (TLR9). Accelerating antigen-specific immune responses by 5-500-fold was shown when they are in close physical contact with immunogen (1-3). While human trials have yielded promising results (4, 5), clinical use of free CpG ODNs still faces several challenges which limit their effectiveness. One of the limiting factors in the success of oligonucleotide-based immunotherapeutics is rapid degradation of unmodified ODNs phosphorothioate (PO CpG) within the body. This problem is diminished by some modifications such as replacement of non-bridging oxygen with sulfur in phosphate linkages to prepare nuclease-resistant phosphorothioate analogs (PS CpG) (6). Despite backbone stabilization of CpG ODNs, PS-modiﬁed ODNs are still susceptible to nuclease degradation, although at a lower rate (7). Moreover, phosphorothioate modiﬁcation is associated with inherent disadvantages including non-sequence speciﬁc toxicity, unfavorable pharmacokinetic (PK) and bio-distribution (BD), poor cellular uptake, and lack of speciﬁcity for target cells (8-11). Administration of high doses of PS CpG has been demonstrated to result in a signiﬁcant acute toxicity in primates due to transient complement activation and other hemodynamic changes which, in extreme cases, may result in cardiovascular collapse and death (7). In addition, PS CpG causes long term severe side effects in mice such as induction of arthritis (12), transient splenomegaly (13), lymphoid follicle destruction (14), and PS CpG-speciﬁc IgM production (15) depending on the CG sequence and backbone modiﬁcation. There are also evidences that PS-modiﬁed ODNs do not closely mimic the interaction of natural PO CpG with TLR9 (10, 16-18). Based on the mentioned reasons and more importantly the lower price of PO CpG compared with PS CpG, we chose PO CpG as the main adjuvant and protect it by entrapment into the liposomes. 

One strategy to protect and extend the activity of PO CpG is encapsulation of CpG ODNs into the liposomes (19, 20). Moreover, liposomes have been used as a delivery vehicle to provide a close association of CpG ODNs with antigens, and enhance the immune responses (19). Lipid based delivery systems are also developed to alter their pharmacokinetic characteristics and enhance immune cell targeting and facilitate intracellular uptake (21).

Induction of cell-mediated immune (CMI) response to poorly immunogenic Ags is possible through encapsulation of Ags into the liposomes (22). Liposomes are lipid based delivery systems that have been used widely to deliver drugs, peptides, proteins, and DNA because of their appropriate properties such as inducing immune response and being safe and biodegradable (23, 24). The structure’s properties such as rigidity, surface charge, and epitope density are efficient in the induced immune response (25). In addition, liposomes make association between antigen and immune-adjuvant to enhance generated immune response (1, 26). 

*Leishmania*, a parasite transmitted by the bite of the sand fly, causes a group of diseases ranging from a self-healing cutaneous lesion (CL) to potentially fatal visceral form of disease, known as leishmaniasis. It was well established that people were infected with cutaneous leishmaniasis, naturally, or with leishmanization, won’t be infected with the disease again. Protection against leishmaniasis is associated with development of a Th1 type of immune response and activation of CD8^+^ T cell population (27, 28). First generation of *Leishmania* candidate vaccines consisting of killed *Leishmania* or parasite fractions have been developed based on this fact. However, they induced only limited prophylactic efficacy and stopped in the phase III of clinical trials mainly due to lack of an appropriate adjuvant (29-33). SLA, as a first generation vaccine, composed of most of the parasite’s soluble antigens provides a wider range of potentially protective epitopes and induces better protection than any of parasite antigens alone (34, 35). We have previously assessed the role of CpG ODNs in enhancement of immune response against two recombinant antigens (rgp63 or rLmSTI1), when entrapped into the liposomes (3, 36). However, considering the promising results obtained from *Leishmania* crude vaccines (37), SLA was used in this study as a model of first generation vaccine with PS or PO CpG either in free or liposomal form to immunize BALB/c mice against leishmaniasis.

## Materials and Methods

***Animals, parasites, SLA, and CpG ODNs***

Female BALB/c mice, 6–8 weeks old, were purchased from Pasteur Institute (Tehran, Iran). The mice were maintained in the animal house of Pharmaceutical Research Centre and fed with tap water and laboratory pellet chow (Khorassan Javane Co., Mashhad, Iran). Animals were housed in a colony room 12/12 hr light/dark cycle at 21 °C with free access to water and food. Experiments were carried out according to the Ethical Committee Acts (Education Office dated March 31, 2010; proposal code 88527) of the Mashhad University of Medical Sciences, Mashhad, Iran.

*Leishmania major* strain (MRHO/IR/75/ER) used in this experiment is the one which was used to prepare experimental *Leishmania* vaccine, leishmanin and leishmanization (37, 38).

SLA preparation was carried out using the protocol developed by Scott *et al *(39) with minor modifications. Briefly, the parasites were harvested at stationary phase and washed for 4 times using HEPES buffer (10 mM, pH 7.5). The number of promastigotes was adjusted to 1.2×10^9^/mL in buffer solution containing enzyme inhibitor cocktail, 50 µl/ml (Sigma, St. Louis, USA), and then the preparation was incubated in ice-water bath for 10 min and the parasites were lysed using freeze-thaw method followed by probe sonication (soniprep-150, MSE, UK) in an ice bath at 4 °C with 20 pulses of 15 sec each at medium amplitude. The supernatant of centrifuged lysate parasites was collected, dialyzed against buffer solution, sterilized using a 0.22 µm membrane, and stored at -70 °C until use. The protein concentration of the preparation was determined using BCA protein assay method (Thermo Scientific, USA).

Two type of the CpG ODNs (Microsynth, Switzerland) were used in this study which contains two CpG motifs, 20-mer termed 1826 (5´- ACG ACG TT-3´) with a nuclease-resistant phosphorothioate backbone (PS CpG ODN) and natural phosphodiester backbone (PO CpG ODN), with known Th1 type immunostimulatory effects on murine model (40, 41).

***Liposomes preparation and characterization***

Liposomes containing SLA and CpG ODNs were prepared using lipid film preparation method. Briefly, the lipid phase consisting of 20 µmol/ml DSPC (Avanti Polar lipids, USA) and 10 µmol/ml cholesterol (Avanti Polar lipids, USA) was dissolved in chloroform in a sterile tube. The solvent was then removed using rotary evaporator (Hettich, Germany) resulting in deposition of a thin lipid ﬁlm on the tube’s wall. The lipid ﬁlm was then freeze – dried (TAITEC, Japan) overnight to ensure complete removal of the solvent. The lipid ﬁlm was then hydrated and dispersed in sterile buffer containing SLA (1 mg/ml) and CpG ODNs (200 µg/ml) at 65 °C. The resulting multilamellar vesicles (MLVs) were converted to large unilamellar vesicles (LUVs) using a bath sonicator (Branson 5510, USA). Then, vesicles size was reduced to 200 nm by a mini-extruder (Avestin, Canada). To prepare liposomes containing only SLA (i.e. Lip-SLA), the same procedure was followed except the CpG ODNs was omitted. 

Particle size analyzer (Nano-ZS, Malvern, UK) was used to estimate the mean diameter and zeta potential of the liposomes. The concentration of SLA encapsulated in liposomes was determined using BCA protein assay kit (Thermo Scientific, USA). Spectrophotometry was used to determine the concentration of CpG ODNs encapsulated in liposomes by using their absorption at 260 nm. Dialysis method (cut-off 300 kD) has been used for separation of entrapped SLA and CpG OND from their free forms. 

***Immunization of BALB/c mice***

Different groups of mice (ten per group) were subcutaneously (SC) immunized in their left hind footpad 3 times in 3-week intervals with one of the following formulations: HEPES buffer (HEPES 10 mM, Sucrose 10%, pH 7.5), SLA (50 µg SLA/50 µl buffer/mouse), Lip-SLA (50 µg SLA/50 µl liposome/mouse), Lip-SLA-PS CpG (50 µg SLA-10 µg CpG ODNs/50 µl liposome/mouse), Lip-SLA-PO CpG (50 µg SLA-10 µg CpG ODNs/50 µl liposome/mouse), SLA (50 µg SLA/50 µl buffer/mouse) plus PS CpG ODN in buffer (10 µg CpG ODNs/50 µl buffer/mouse), SLA (50 µg SLA/50 µl buffer/mouse) plus PO CpG ODN in buffer (10 µg CpG ODNs/50 µl buffer/mouse). 

***Challenge with L. major promastigotes***

The immunized mice (seven per group) were challenged SC in the left footpad with 1×10^6^ *L. major* promastigotes harvested at stationary phase in 50 µl volume, at week 2 after the last immunization booster. Lesion development was recorded in each mouse by measurement of footpad swelling using a metric caliper (Mitutoyo Measuring Instruments, Japan). Grading of lesion size was done by subtracting the thickness of the uninfected contralateral footpad from that of the infected one.

***Quantitative parasite burden in spleen***

The number of viable *L. major* parasites in the spleen of mice was estimated by a limiting dilution assay method as described previously (42). Briefly, the mice were sacriﬁced at week 7 post-challenge; the spleens were aseptically removed and homogenized in RPMI 1640 supplemented with 10% v/v heat inactivated (Eurobio, France), glutamine, 100 U/ml of penicillin, and 100 µg/ml of streptomycin sulfate (RPMI-). The homogenate was diluted with the media in 8 serial 10-fold dilutions and then was placed in each well of ﬂ. -bottom 96-well microtiter plates (Nunc, Denmark) containing solid layer of rabbit blood agar in tetraplicate and incubated at 26±1 °C for 7-10 days. The number of viable parasite per spleen was determined by ELIDA software, a statistical method for limiting dilution assay (43).

***Antibody isotype assay***

Blood samples were collected from mice before and at week 7 after challenge and the sera were isolated and kept frozen until being used to assess anti-SLA IgG total, IgG1, and IgG2a antibodies by ELISA method as described before (42). Briefly, 96-well micro titer plates (Nunc, Denmark) were coated with 50 µl of 10 µg/ml of SLA in PBS buffer (0.01 M, pH 7.3) overnight at 4 °C. Plates were washed and then were blocked by adding 200 µl per well of 1% of bovine serum albumin in PBS-Tween and incubated for 1 hr at 37 °C. Serum samples were diluted to 1:200, 1:2,000, or 1:20,000 with PBS–Tween and applied to the plates. The plates were then treated with -rabbit anti-mouse IgG isotype according to the manufacturer’s instructions (Zymed Laboratories Inc., USA). Optical density (OD) was determined at 450 nm using 630 nm as the reference wavelength.

***In vitro spleen cells response***

Three mice from each group were sacriﬁced at week 2 after the last booster injection (before challenge) and the spleens were aseptically removed. Mononuclear cells were isolated by Ficoll-Hypaque density gradient centrifugation method from spleen cell suspension. The cells were washed and resuspended in RPMI 1640- and seeded at 2×10^6^ cells/ml in 96-well ﬂat-bottom plates (Nunc, Denmark). The cells were stimulated *in vitro* with either SLA (10 µg/ml), Con A (2.5 µg/ml), or medium alone and incubated at 37 °C in 5% CO_2_ for 72 hr. The culture supernatants were collected and the levels of IL-4 and IFN-γ were checked using ELISA method according to the manufacturer’s instructions (MabTech, Sweden).

**Figure 1:**
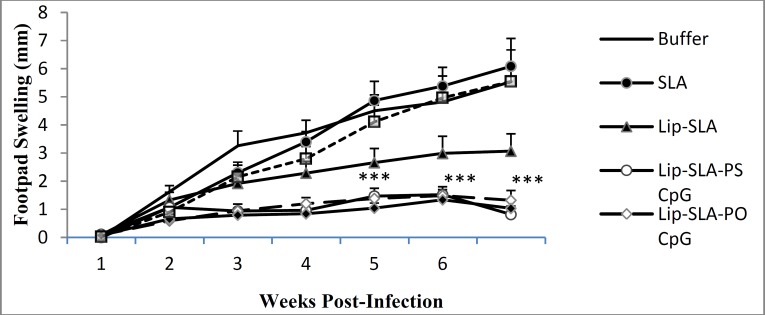
Footpad swelling in BALB/c mice immunized SC, 3 times in 3 weeks intervals, with buffer, SLA, Lip-SLA, Lip-SLA-PS CpG, Lip-SLA-PO CpG, SLA+PS CpG, or SLA+PO CpG after challenge with virulent *Leishmania major* promastigotes. The mice were challenged in the left footpad with 10^6^ *Leishmania major* promastigotes, 2 weeks after the last booster. The footpad thickness of mice was then measured on both footpads for 7 weeks. Each point represents the average increase in footpad thickness±SEM (n = 5). ***P< 0.001 indicates that the values of marked immunized mice are significantly different from those received HEPES buffer.

***Statistical analysis***

One-way ANOVA statistical test was used to assess the significance of the differences among various groups. In the case of signiﬁcant F value, Tukey–Kramer multiple comparisons test was carried out as a post-test to compare the means in different groups of mice. Results with *P*< 0.05 were considered to be statistically signiﬁcant.

## Results

***Liposome characterization***

As shown in Table 1, liposomes used in this study were 230-310 nm in size as calculated by particle size analyzer with a mean diameter of 272.83±29.35 and 296.60±24.17 nm (n= 3) and the zeta potential of -23.20±1.49 and-33.57±1.94 mV for Lip-SLA-PS CpG and Lip-SLA-PO CpG, respectively. The amount of SLA entrapment measured by BCA protein assay was 52±5% (n= 3). The encapsulated PO CpG and PS CpG percent in liposomes were 9.8±1.2% and 9.2±0.8% (n= 3), respectively.

***Challenge results***

Lesion development was monitored by weekly measurement of footpad thickness (Figure 1). The lesion size progressed at a more rapid rate in control groups which received either buffer or SLA alone compared with mice immunized with Lip-SLA-PS CpG or Lip-SLA-PO CpG at week 4 after challenge and after that (*P*< 0.001). There was no significant differences between the groups which received Lip-SLA-PS CpG and those immunized with Lip-SLA-PO CpG. The lesion size in mice immunized with Lip-SLA-PO CpG was significantly (*P*< 0.05) smaller than those received SLA+PO CpG at week 4 after challenge and even more at week 6 (*P*< 0.01). The lesion size in mice immunized with buffer, SLA alone or SLA+PO CpG was progressed continuously and no protection was observed in these groups and the footpad swelling reached a plateau after 6 weeks but interestingly, the groups that received Lip-SLA-PS CpG or Lip-SLA-PO CpG showed significant reduction in the swelling size from week 5 to 6 post-challenge.

***Splenic parasite burden***

**Figure 2:**
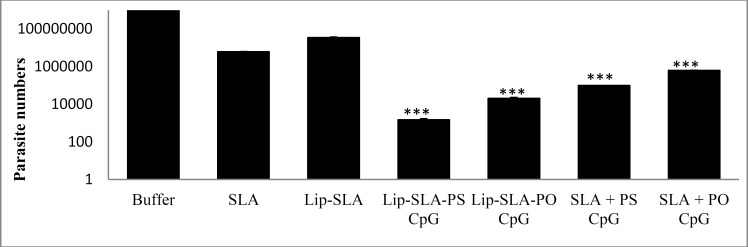
Spleen parasite burden in BALB/c mice immunized SC, 3 times in 3 weeks interval, with buffer, SLA, Lip-SLA, Lip-SLA-PS CpG, Lip-SLA-PO CpG, SLA+PS CpG, or SLA + PO CpG after challenge with *Leishmania major* promastigotes. A limiting dilution analysis was performed at week 7 after the challenge on the spleen of individual mice and cultured in quadruplicate in serial of 8-fold dilutions. The number of viable parasites per spleen was determined using ELIDA software. The bar represents the average score±SD (n= 3)

The number of viable *L. major* was determined in the spleen of different groups of mice at week 7 after challenge (Figure 2). The estimated number of parasites in mice’s spleen which received buffer, SLA, or Lip-SLA was higher than the groups (*P*< 0.001) received Lip-SLA-PS CpG or Lip-SLA-PO CpG. Interestingly, no difference was observed between these two groups. However, there was no difference between the mice immunized with Lip-SLA-PS CpG or Lip-SLA-PO CpG and those received SLA+PS CpG or SLA+PO CpG.

***Antibody response***

**Figure 3:**
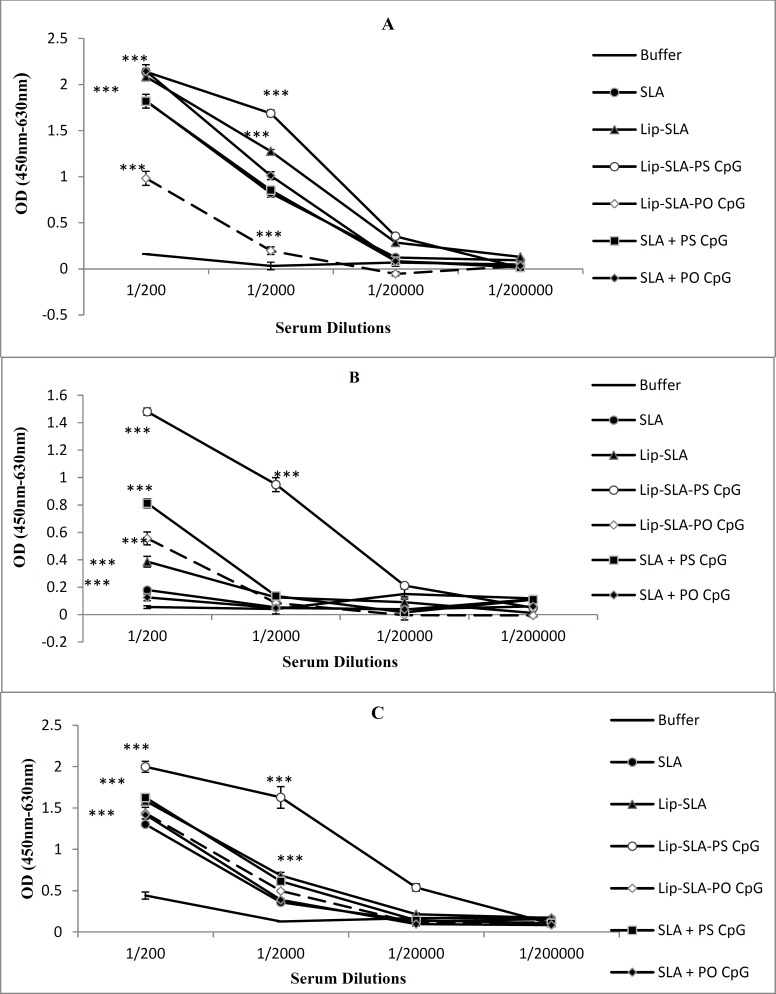
Levels of anti-SLA IgG1 (A), IgG2a (B), and IgG (C) in pooled sera of BALB/c mice immunized SC, 3 times in 3 week intervals, with buffer, SLA, Lip-SLA, Lip-SLA-PS CpG, Lip-SLA-PO CpG, SLA+PS CpG or SLA+PO CpG ODN(O). Blood samples were collected from the mice 2 weeks after the last booster. The anti-SLA IgG1, IgG2a, and IgG levels were assessed using ELISA method. The assays were performed in 200, 2000, 20000, and 200000 fold dilution for each serum sample. Values are the Mean±SD. ***P<0.001 indicates that the values of marked immunized mice are significantly different from those received buffer

**Figure 4:**
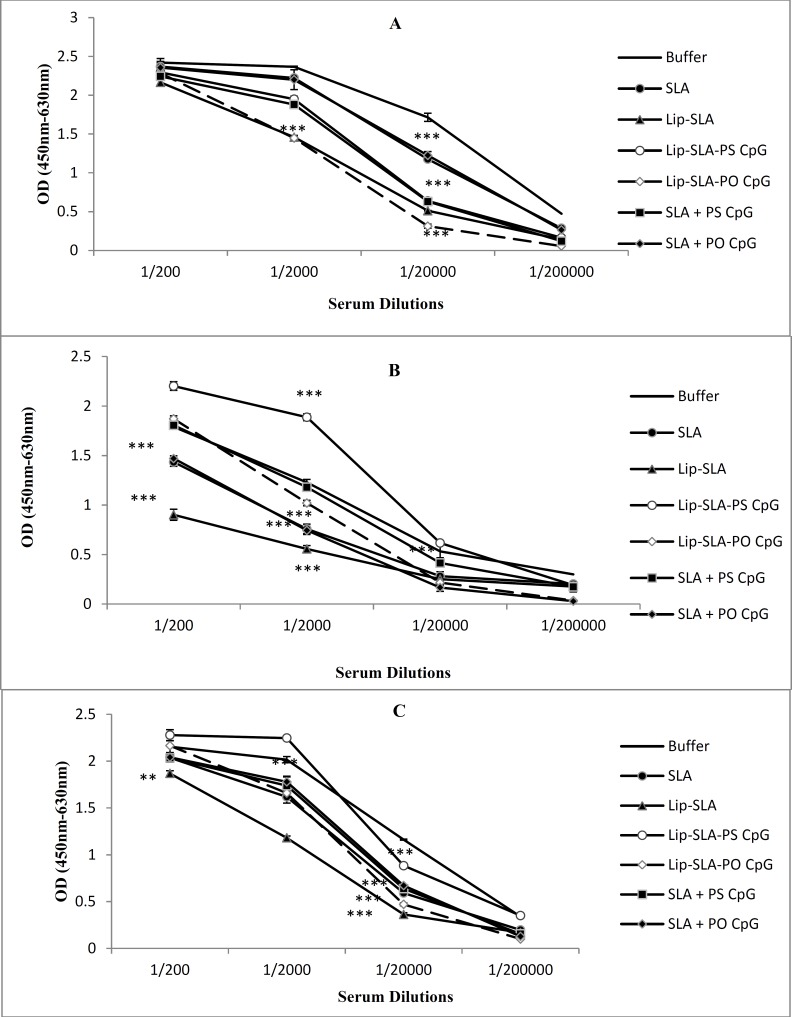
Levels of anti-SLA IgG1 (A), IgG2a (B), and IgG (C) in pooled sera of BALB/c mice immunized SC, three times in 3 week intervals, with buffer, SLA, Lip-SLA, Lip-SLA-PS CpG, Lip-SLA-PO CpG, SLA+PS CpG or SLA+PO CpG. Blood samples were collected from the mice 7 weeks after challenge. The anti-SLA IgG1, IgG2a and IgG levels were assessed using ELISA method. The assays were performed in 200, 2000, 20000, and 200000 fold dilution for each serum sample. Values are the Mean±SD. ***P< 0.001 and **P< 0.01 indicate that the values of marked immunized mice are significantly different from those received buffer

In order to determine the type of generated immune response, the anti-SLA IgG antibodies and IgG1 and IgG2a subclasses were titrated before (Figures 3A, 3B and 3C) and after (Figures. 4A, 4B and 4C) the challenge. 

The sera of mice immunized with Lip-SLA-PS CpG or Lip-SLA-PO CpG before challenge showed a signiﬁcantly (*P*< 0.001) higher levels of IgG1, IgG2a, and IgG total antibodies compared with the control groups (buffer or SLA) with just an exception in IgG1 titer so that the level of IgG1 antibody in sera of mice immunized with Lip-SLA-PO CpG was lower than SLA in soluble form. The level of IgG1, IgG2a, and IgG in the group of mice immunized with Lip-SLA-PS CpG were higher than groups immunized with Lip-SLA-PO CpG (*P*< 0.001) or SLA+PS CpG (*P*< 0.001). Comparison between groups received Lip-SLA-PO CpG or SLA+PO CpG showed significant differences (*P*< 0.001) in all of evaluated antibody isotypes. The level of IgG2a antibody in mice immunized with Lip-SLA-PO CpG was significantly (*P*< 0.001) higher than those received SLA+PO CpG, although there was no significant difference in IgG titer. Interestingly, the level of IgG1 antibody in groups received SLA+PO CpG was higher than those received Lip-SLA-PO CpG. 

**Table 1:** Particle size, polydispersity index (PDI), and surface charge of various liposomal formulations. Results denote mean±SD (n=3)

Samples	Size (nm)	Polydispersity index	Zeta potential (mV)	Zeta deviation
Empty liposome	230.43±65.32	0.289±0.14	-6.54±1.31	5.40±1.06
Lip-SLA	306.36±23.61	0.233±0.04	-18.13±0.97	4.95±0.45
Lip-SLA-PS CpG	272.83±29.35	0.207±0.80	-23.20±1.49	4.75±0.22
Lip-SLA-PO CpG	296.60±24.17	0.275±0.12	-33.57±1.94	7.31±0.32

Challenge with *L. major* promastigotes induced higher IgG1, IgG2a, and IgG antibody levels in almost all the group of mice compared with before challenge (Figures 4A, 4B and 4C). The sera of mice immunized with Lip-SLA-PS CpG or Lip-SLA-PO CpG showed a signiﬁcantly (*P*< 0.001) higher levels of IgG2a and IgG total antibodies and lower levels (*P*< 0.001) of IgG1 antibody compared with the control groups. The levels of IgG1, IgG2a, and IgG total in the group received Lip-SLA-PS CpG was significantly higher than group received Lip-SLA-PO CpG (*P*< 0.001). The mice immunized with Lip-SLA-PO CpG showed higher levels of IgG2a and IgG total and lower level of IgG1 than those immunized with SLA+PO CpG (*P*< 0.001). The ratio of IgG2a/IgG1 in sera of mice immunized with Lip-SLA-CpG was higher than all the other groups and even higher than this ratio before challenge (~650 before challenge *vs.* 880 after challenge).

***In vitro cytokine production by splenocytes***

**Figure 5:**
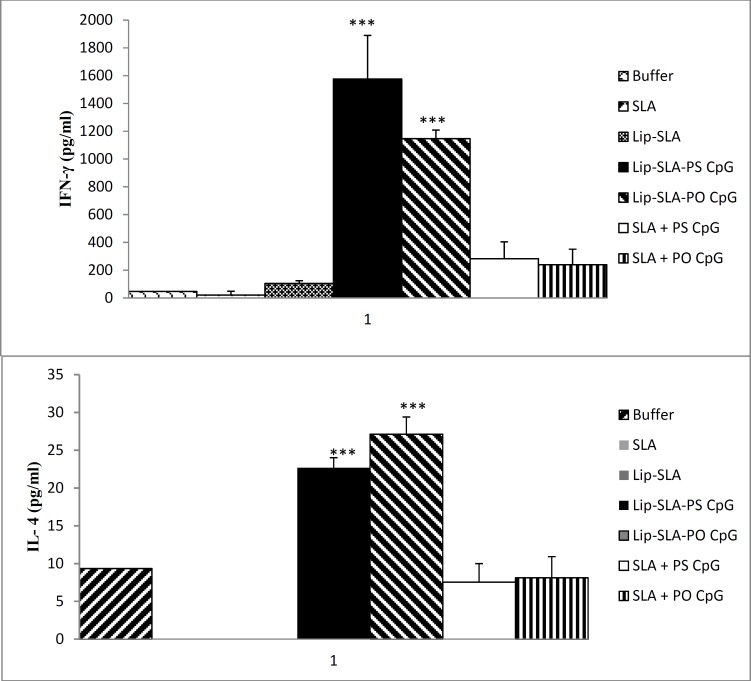
Cytokine levels in immunized mice at week 2 after the last booster injection. Mononuclear splenocytes were cultured in the presence of SLA (10 µg/ml) and the level of IFN-γ (A) or IL-4 (B) in the culture supernatants were detected using ELISA method. Results are shown as the Mean±SEM (n= 3)

The supernatant of cultured splenocytes given from mice sacrificed 2 weeks after the last booster restimulated *in vitro* with SLA and was analyzed to determine the level of IFN-γ and IL-4 and cytokines indicative of Th1 and Th2 response, respectively. The results showed that the group of mice immunized with Lip-SLA produced the highest amount of IFN-γ compared with the control groups but the lowest amount compared with all the groups which received CpG ODNs (*P*< 0.001) either in free or liposomal form. Moreover, the level of IFN-γ in mice immunized with Lip-SLA-PS CpG or Lip-SLA-PO CpG showed significantly higher level than SLA+PS CpG or SLA+PO CpG, respectively (*P*< 0.001). Surprisingly, there was no significant difference between the groups received Lip-SLA-PS CpG or Lip-SLA-PO CpG Figure 5A). The same results were observed in IL-4 titration in liposomal groups containing CpG ODNs. There was no significant difference between the mice immunized with Lip-SLA and those received the control groups (Figure 5B). 

## Discussion

In the current study, the role of liposomes in the protection of PO CpG ODN when administered *in vivo *was assessed and compared with PS CpG ODN. Moreover, their adjuvanticity effect was studied against leishmaniasis in murine model.

Interaction between CpG ODNs and TLR9 which presents in intracellular compartment of APCs (44-46), begins an immunostimulatory cascade leading to the maturation, differentiation, and proliferation of multiple immune cells (47, 48). The severe susceptibility of natural phosphodiester (PO) form of CpG to nuclease degradation makes them partially inactive in the free form and limits their use *in vivo *(49, 50). Replacement of a non-bridging oxygen atom in the PO bond with a sulfur atom (phosphorothioate or PS) is one way to solve this problem (49). However, the modified CpG ODNs created some other problems that has been raised after using this component *in vivo *(21). Therefore, using delivery systems to prevent *in vivo *degradation of PO CpG ODNs may be a superior way to increase PO CpG ODNs’ adjuvanticity.

Liposomes are lipid based delivery systems that has been used widely to deliver drugs, peptides, proteins, and DNA because of their appropriate properties such as being safe and biodegradable (23, 24). Liposomes also channel protein and peptide antigens into the major histocompatibility complex class II ( II) pathways of APCs, resulting in enhanced antibody and antigen-speciﬁc T-cell proliferative response based on their formulations (51). Liposomes have been shown to be an effective delivery system to improve adjuvanticity of CpG ODNs. It seems that CpG ODN in free form induces protection for a shorter time, but liposomal CpG ODN induces longer protection (52). More importantly, when CpG ODN is used in liposomal form, the proximity of adjuvant to antigen is maintained (2). 

In order to assess the protection rate, footpad swelling induced by *L. major* was measured in immunized mice and compared with that of the control groups. Our results indicated the superiority of liposomal form of CpG ODNs in order to stimulate a Th1 immune response. An improvement of adjuvanticity of CpG ODNs was previously reported by encapsulation of CpG ODNs in liposomes (53, 54). However, PS CpG in their free forms showed significantly higher protection rate than PO CpG as shown in Figures 1 and 2. The results showed a more potent and durable protection with soluble PS CpG ODN than in obsess of it that was in agreement with the other studies (55-57).

The results of antibody assay in groups received Lip-SLA-PO CpG or SLA+PO CpG showed a significant difference in IgG2a and gG1 subclasses. The level of IgG2a antibody in mice immunized with Lip-SLA-PO CpG was significantly (*P*< 0.001) higher than those received SLA+PO CpG, but it was opposite regarding IgG1 level. The mice immunized with Lip-SLA-PO CpG showed the same response as those received Lip-SLA-PO CpG and stimulated Th1 type of immune response but it was not true for mice which were immunized with SLA+PO CpG. On the other hand, PO CpG in free form cannot induce Th1 type of immune response because of its instability.

The results of cytokine assay showed that the level of IFN-γ in the supernatant of cultured splenocytes of mice immunized with Lip-SLA-PO CpG or Lip-SLA-PS CpG was higher than the other immunized groups. Surprisingly, the IL-4 level was also high in these groups but it was very low (under detection limit) in groups immunized with Lip-SLA or SLA. At APCs level, CpG ODNs augment both activation and maturation of DC as well as the induction of proinflammatory cytokines (58). Thus, the endogenous production of IL-12, IL-18, and other soluble mediators from activated DC induced by CpG ODNs are likely to result in a more physiologic cognate interaction between DC and T cell, which results in qualitatively and quantitatively different types of CD4+ and CD8+ T-cell response (59). Based on the cytokine assay results, it can be concluded that the presence of CpG ODN in liposomal form induced a mixed Th1/Th2 response.

The idea of using SLA was based on a theory that says a cocktail Ag vaccine, which contains most of parasite antigens, provides a wider range of potentially protective epitopes than any of parasite’s antigens alone (35). In comparison with recombinant *Leishmania* antigens such as gp63 or LAg, SLA has already shown better immune response when used in liposomal form (34). However, the results of the current study showed that SLA entrapped in liposome doesn’t induce efficient protection because there was a significant difference between the mice received Lip-SLA and those received liposomal SLA containing CpG ODNs that might be due to different liposome formulations in these studies. Neutral and positively charged liposomes were prepared with egg lecithin and cholesterol or with egg lecithin, cholesterol, and stearylamine, respectively. However, in this study, DSPC has been chosen as a main liposome’s lipid based on its neutral charged and high transition temperature (T*c*= 54 ºC). The role of phospholipids in extent of induced immune response against leishmaniasis was assessed previously and results showed that DSPC is more efficient than DPPC, DMPC, or EPC regarding stimulation of Th1 type of immune response (60, 61). Liposomes with high *Tc* have greater membrane rigidity *in vivo* and therefore entrap solutes (e.g. antigens) and antigens remain with the carrier for a longer period of time (61). 

The effect of bilayer charge on the cellular uptake of liposomes is a matter of considerable discussion (62-64). Some researchers suggest positively charged liposomes because negative charge of cell surface of APCs makes better uptake for positively charged liposomes (63, 64) and some studies showed better uptake with negatively charged liposomes (62). In our previous study, an efficient cationic liposomal protein-based vaccine against *L. major* infection was developed using SLA and PS CpG ODNs. The prepared formulation was appropriate to induce Th1 type of immune response against leishmaniasis (65). However, due to partial toxicity of cationic lipids, neutral lipid was exploited in the current study. As shown in Table 1, the presence of SLA and/or CpG ODNs produced a negative charge on the surface of neutral liposomes which was similar to parasites surface charge. The same results have been reported in our previous study that shows SLA has a negative charge (65). Lipophosphoglycans (LPGs) is the major cell surface macromolecule and plays a key role in determining parasite virulence and survival in the mammalian macrophage. LPG-like molecules are also expressed on the cell surface of the amastigotes and may be necessary for parasite survival in the macrophage phagolysosome compartment (66). Sialic acids associated with glycoproteins, glycolipids, and phosphate groups in LPGs are the major components responsible for the net negative surface charge of the trypanosomatids such as *Leishmania* (67).

## Conclusions

The current data pointed out that there is no significant difference between PS CpG and PO CpG in terms of their ability to induce Th1 response when encapsulated into the liposomes. Therefore, our results suggested that liposomal form of PO CpG might be used instead of PS CpG in future vaccine formulations as an efficient adjuvant. In addition, the promising role of CpG ODNs and neutral DSPC liposomes as adjuvants to enhance stronger immune response against SLA was confirmed. 
